# MR imaging findings of primary ovarian carcinoid: A novel finding of T1 hyperintense solid tissue

**DOI:** 10.1016/j.radcr.2024.03.086

**Published:** 2024-04-24

**Authors:** Shinya Fujii, Chie Inoue, Hiroto Yunaga, Takuro Gonda, Jun Makishima, Ryoya Ochiai, Daisuke Yamaji, Kanae Ozaki

**Affiliations:** a Division of Radiology, Department of Multidisciplinary Internal Medicine, Faculty of Medicine, Tottori University, Yonago, Japan; b Department of Radiology, Tottori Prefectural Central Hospital, Tottori, Japan; c Department of Pathology, Faculty of Medicine, Tottori University, Yonago, Japan

**Keywords:** Magnetic resonance imaging, Ovary, Carcinoid

## Abstract

Ovarian carcinoid is a rare well-differentiated neuroendocrine tumor resembling those arising in the gastrointestinal tract. We present a case of ovarian carcinoid with magnetic resonance imaging (MRI) findings. A 50-year-old woman with genital bleeding and severe constipation was referred to our hospital. On MR imaging, a left ovarian tumor showed iso to high signal intensity on T1-weighted images (T1WI), relatively low signal intensity on T2WI, and slightly high signal intensity on diffusion-weighted images. Additionally, the tumor demonstrated early and delayed strong contrast enhancement on dynamic contrast-enhanced images. The tumor was pathologically diagnosed with ovarian strumal carcinoid. High signal intensity on T1WI should be recognized as the MRI findings in ovarian carcinoids.

## Introduction

Ovarian carcinoid is a well-differentiated neuroendocrine tumor resembling those arising in the gastrointestinal tract [1]. It accounts for <0.1% of all ovarian neoplasms and for <5% of all carcinoid tumors [2]. Magnetic resonance imaging (MRI) findings of primary ovarian carcinoids are limited due to their rarity. Herein, we report the MRI findings of a case of ovarian carcinoid, particularly high signal intensity on T1-weighted imaging (T1WI).

## Case report

A 50-year-old woman (G3 P2 AA1) was referred to our hospital for genital bleeding. Laboratory findings revealed mild anemia and elevated inflammatory markers. Serum tumor markers were not elevated. The patient had also severe constipation. A left ovarian mass was noted on vaginal ultrasonography. MRI was performed for further examination.

**Fig. 1:**
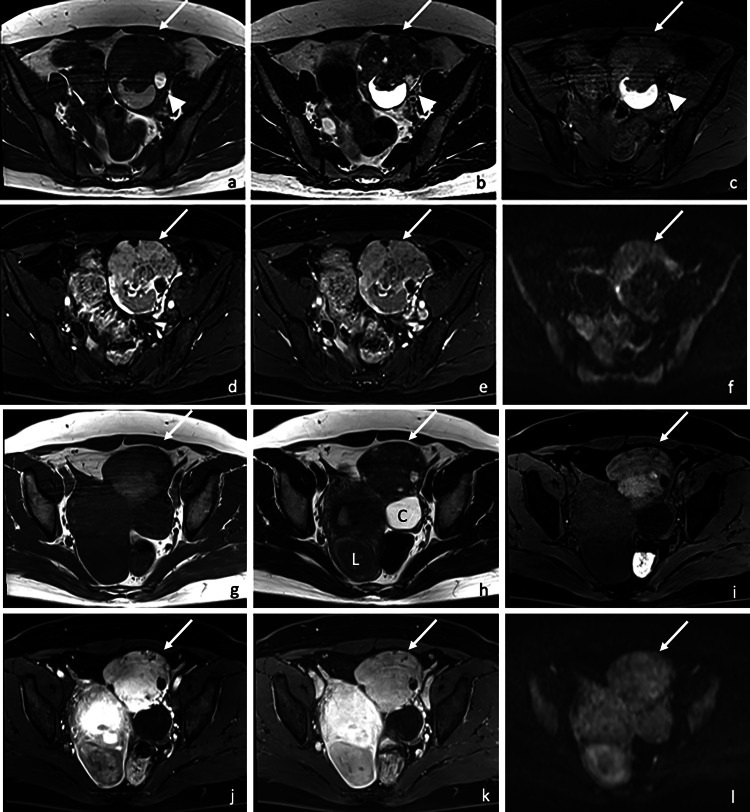
A 50-year-old woman with a histologically proven ovarian carcinoid (a and g) T1-weighted imaging (T1WI), (b and h) T2WI, (c-e) (i-k) pre-, early-, and late-phase dynamic contrast-enhanced imaging, (f and l) diffusion weighted imaging (DWI). Magnetic resonance images demonstrate a left ovarian tumor. A small part of the cystic areas shows high signal intensity on both T1WI and T2WI, and low signal intensity on fat-suppressed T1WI, indicating a fatty component (a-c arrow heads). Another part shows high signal intensity on both T1WI and T2WI without signal suppression on fat-suppressed T1WI, suggestive of viscous fluid. The solid component (arrows) shows iso to high intensity on T1WI (a, g), inhomogeneous iso to relatively low signal intensity on T2WI (b, h) compared with the myometrium, and mild high signal intensity on DWI (f, l) with low signal intensity on ADC map (not shown). The solid component shows strong contrast enhancement on early (d, j) and late phase (e, k) dynamic contrast-enhanced images. A functional ovarian cyst is also observed at the posterior side of the tumor (h, letter C). A leiomyoma is observed (h, letter L).

MRI demonstrated a 9 cm left ovarian mass comprising solid and cystic areas. A small part of the cystic areas showed high signal intensity on both T1WI and T2WI, and low signal intensity on fat-suppressed T1WI, indicating a fatty component. Another part showed high signal intensity on both T1WI and T2WI without signal suppression on fat-suppressed T1WI, suggestive of viscous fluid. The solid area showed iso to relatively high signal intensity on T1WI, and inhomogeneous iso to relatively low signal intensity on T2WI compared with the outer myometrium (Fig. 1). The solid component showed early intense contrast enhancement on dynamic contrast-enhanced (DCE) images (Fig. 1). On diffusion-weighted images (DWI) with b value of 1000, the mass demonstrated slightly high signal intensity (Fig. 1) despite the decreased apparent diffusion coefficient (ADC) values (0.83 × 10^−3^ mm^2^/s).

**Fig. 2:**
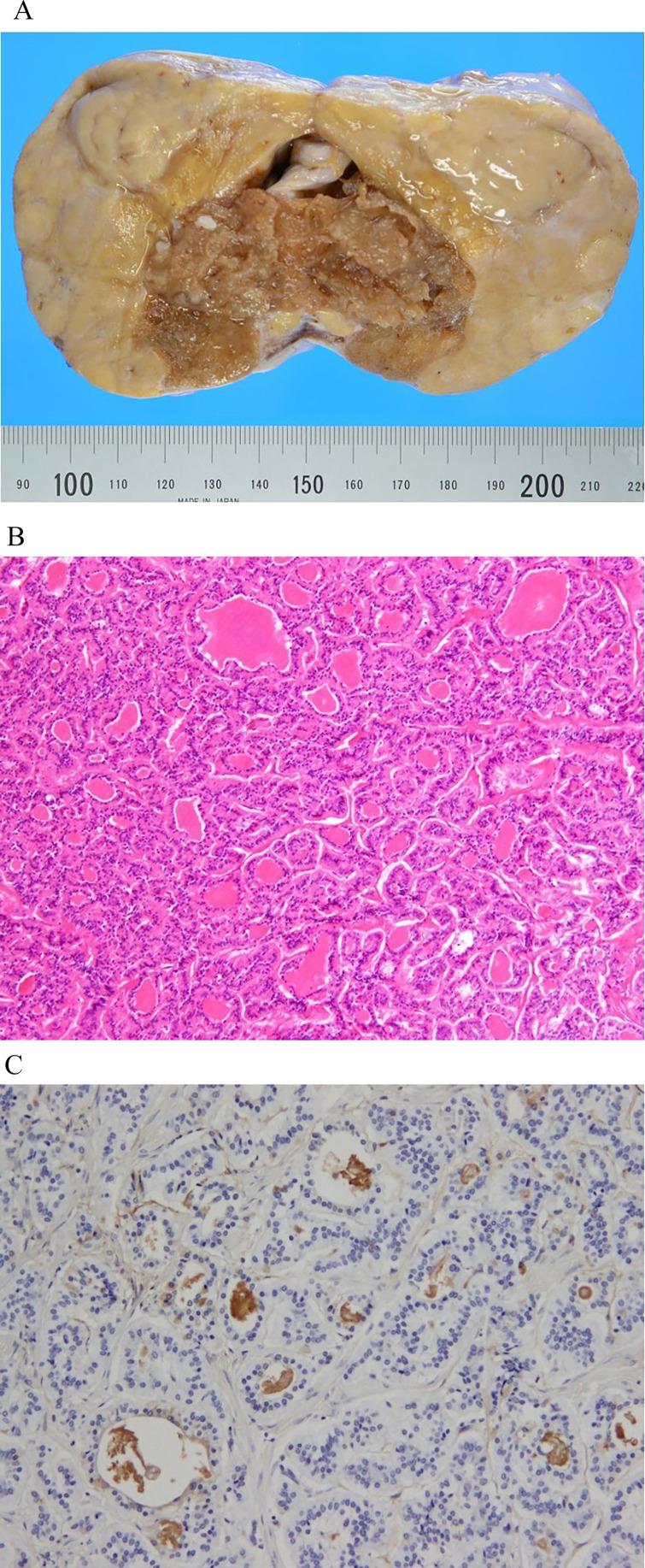
(A) Gross specimen, (B) Hematoxylin and eosin (H & E) stain high-power field, (C) immunohistochemical for thyroglobulin high-power field. The tumor is split open. Macroscopic examination reveals an elastic hard solid mass, containing fat and a brownish thyroid-like tissue (A). A trabecular or ribbon-like structure comprising cuboidal to columnar cells is admixed with a follicular structure filled with eosinophilic colloid materials. No necrosis or mitosis is observed (B). The colloid material in the follicular structures is positive for thyroglobulin on immunohistochemistry (C).

Bilateral salpingo-oophorectomy was performed. Macroscopic examination revealed an elastic hard solid mass, containing fat and a brownish thyroid-like tissue (Fig. 2). No obvious necrosis was observed. On microscopic examination, the solid component comprised cuboidal to columnar tumor cells with oval to round nuclei presenting a trabecular, ribbon-like, or follicular arrangement (Fig. 2). Insular growth was focally observed. The tumor component was merged with thyroid-like follicular tissues. The tumor cells were positive for chromogranin A, synaptophysin, and CD56, positive for CEA, and negative for calcitonin on immunohistochemistry. The colloid component of the follicles was positive for thyroglobulin, suggesting that these follicular structures were derived from teratomatous thyroid tissue (Fig. 2). The Ki-67 labeling index of the tumor cells was <3%, and no mitosis was observed. The tumor was finally diagnosed as strumal carcinoid. After the resection, her severe constipation resolved.

## Discussion

Primary ovarian carcinoids are rare. They are commonly seen in postmenopausal women (mean age: 53) [1]. Ovarian carcinoids are subdivided into 4 categories: insular, trabecular, mucinous, and strumal [3]. The most common subtype is insular, followed by strumal [1]. Carcinoids are considered monodermal teratomas arising from neuroendocrine cells within the intestinal-type epithelium of mature cystic teratomas [1]. The carcinoid component of strumal carcinoids is considered to be a malignant transformation of struma ovarii [4]. One-third of the patients with insular carcinoid present with carcinoid syndrome, such as facial flushing, diarrhea, and edema, due to the direct production of serotonin-like substances into the systemic circulation through the ovarian venous system bypassing hepatic deactivation [5]. Additionally, constipation is also known as a symptom of carcinoid syndrome due to the release of peptide YY (PYY) in strumal and trabecular carcinoids. PYY is a gastrointestinal peptide present mainly in the endocrine cells of the distal intestine and inhibits motility [6]. The typical management for ovarian carcinoids is surgical resection. The prognosis is usually excellent, with rare exceptions. In the present case, the patient's severe constipation resolved after resection. Therefore, it was suggested that the tumor produced PYY.

In the present case, the solid component of the tumor showed relatively high signal intensity on T1WI. High signal intensity on T1WI has been reported to be caused by hemorrhage [7] and protein content within the tumors in hepatic and gastrointestinal carcinoids [8]. On the other hand, we consider that relatively high signal intensity on T1WI in ovarian carcinoids may contribute to the colloids in thyroid tissues, which is a viscous fluid. Thus, ovarian carcinoid should be included as a solid tumor with relatively high signal intensity on T1WI.

Additionally, the solid portion of ovarian carcinoid showed relatively low signal intensity on T2WI. The T2 hypointensity has been reported to reflect fibromatous stroma from serotonin produced by the tumor [9]. Moreover, the tumor showed early and delayed strong contrast enhancement on DCE imaging suggesting hypervascularity, which is consistent with a previous study [9]. Further, the tumor demonstrated mild high signal intensity on DWI in spite of the decreased ADC value. Although the reason for the mild high signal intensity is unclear, T2-blackout effect is suggested.

The differential diagnosis was struma ovarii. Struma ovarii usually demonstrates a cystic mass with a solid component. The solid components typically demonstrate thickened walls and rarely mass-like lesions [10]. The solid portions show iso to slightly high signal intensity on T1WI and low to iso signal intensity on T2WI [10]. Regarding DWI, the solid portion has been reported to show relatively high signal intensity on DWI, but high ADC value [11].

In conclusion, we presented a case of ovarian carcinoid with MRI findings. Relatively high signal intensity in the solid component on T1WI should be recognized as the MRI findings in ovarian carcinoids.

## Patient consent

Written informed consent was obtained from the patient prior to submission of this case report.
